# Misinformation and disinformation undermine progress in pediatric research: challenges and solutions

**DOI:** 10.1038/s41390-025-04370-w

**Published:** 2025-10-06

**Authors:** Mark R. Schleiss, Lee Beers, Lisa J. Chamberlain, Sangeeta Hingorani, Henry C. Lee, Scott A. Lorch, Pooja Tandon, Cindy W. Christian

**Affiliations:** 1 Division of Pediatric Infectious Diseases, Department of Pediatrics, University of Minnesota Medical School, Minneapolis, MN USA; 2 Children’s National Research Institute, Washington, DC USA; 3 Department of Pediatrics, Office of Child Health Equity, Stanford University School of Medicine, Palo Alto, CA USA; 4 Department of Pediatrics, Seattle Children’s Research Institute, University of Washington School of Medicine, Seattle, WA USA; 5 Department of Pediatrics, UCSD School of Medicine, La Jolla, CA USA; 6 Department of Pediatrics, Children’s Hospital of Philadelphia, The University of Pennsylvania Perelman School of Medicine, Philadelphia, PA USA; 7 University of Washington Medical School, Seattle Children’s Research Institute, Seattle, WA USA; 8 Department of Pediatrics, Safe Place: The Center for Child Protection and Health, Children’s Hospital of Philadelphia, The University of Pennsylvania Perelman School of Medicine, Philadelphia, PA USA; 9 Academic Pediatric Association, McLean, VA USA; 10 American Pediatric Society, Itasca, IL USA; 11 Society for Pediatric Research, The Woodlands, TX USA; 12 Association of Medical School Department Chairs, McLean, VA USA

## Introduction

Rapidly evolving societal forces in the United States (US) have created a divisive landscape threatening the health of American children through a loss of confidence in biomedical research in the US which undermines seminal achievements in pediatric scientific discovery that have positively affected children’s lives in the past several decades. It is vital that we combat misinformation and disinformation and the negative impact of this evolving trend on public health, policy initiatives, and medical progress. For the purposes of this paper, we use the American Psychological Association’s Consensus Report (https://www.apa.org/pubs/reports/health-misinformation), which defines misinformation as false or inaccurate information (even if well-intended), regardless of how it originated; in contrast, we define disinformation as untrue assertions that are disseminated with deliberate intent to deceive. By undermining confidence in our past accomplishments and promoting false information about the state of child health, both phenomena threaten future innovation and progress in child health research.^1^

## Misinformation and the MAHA report: misplaced priorities and unrecognized urgencies

On February 1, 2025, an executive order established the Make America Healthy Again (MAHA) Commission to report on the current state of child health in the US and was chaired by the Department of Health and Human Services (HHS) Secretary Robert F. Kennedy Jr. On May 22, 2025, the commission’s initial assessment of child health “Make Our Children Healthy Again” was released (https://www.whitehouse.gov/wp-content/uploads/2025/05/WH-The-MAHA-Report-Assessment.pdf). The Commission’s conclusion that “today’s children are the sickest generation in American history…” was attributed to four main drivers of chronic childhood illness (Table 1)^2^: highly processed foods, excessive chemical intake, behavioral health crisis associated with excessive reliance on digital devices, and “over-medicalization” of care. The report’s conclusions are not without merit. Indeed, there is an ongoing crisis in pediatric mental health in the US.^3,4^ The overuse of electronic devices clearly contributes to adverse child health outcomes.^5^ Nutritional issues, especially undernourishment and excessive dietary reliance on highly processed foods, unquestionably impact child physical, cognitive and behavioral development.^4,6,7^ Although these important issues identified by the MAHA report require solutions, focusing what may be excessive emphasis on these issues may divert attention from even more compelling factors that impact child health, as outlined below.

**Table 1:** Key reasons for decline in child health in USA, as assessed by the HHS MAHA report, 2025

MAHA Child Health Report	Reasons Cited by Report
A shift in recent decades to ultra-processed foods as the cornerstone of childhood dietary intake.	The report states that children’s diets are increasingly dominated by ultra-processed foods, which are high in added sugars, unhealthy fats, and refined grains, and low in fiber and essential nutrients.
The cumulative load of chemicals in the environment, and the attendant burden of these chemicals on child health and development.	Concerns are noted about the negative health effects of childhood exposure to environmental chemicals, including pesticides, microplastics, and food additives.
A crisis in child behavior during the “digital age”, including a decline in physical activity and a deterioration in childhood mental health.	The report identifies sedentary lifestyles, increased amounts of screen time, and chronic stress as issues that are contributing to the decline in physical and mental health.
The overmedicalization of children, including the excessive use of prescription medications, unhealthy growth of the childhood vaccine schedule, and the influence of “corporate capture”, in which industry interests dominate and distort pediatric health care, potentially leading to unwarranted access to healthcare services in pediatric practice.	The report cites the overuse of prescription drugs as detrimental to children’s health. It also raises concerns about the safety of medical procedures, including the childhood vaccination schedule.

## Acknowledge pressing needs without erasing recognition of transformative scientific advances in pediatric care

While some concerns outlined in the MAHA pediatric health are clearly supported by evidence, several assertions represent examples of medical misinformation about the state of child health in the US. For example, it has been asserted that US children are experiencing an overall decline in their health compared to previous generations. A 2018 survey found that less than one-third of adults believed that children were physically healthier when compared to their own childhoods.^8^ In reality, the myriad of basic, clinical and translational research accomplishments in pediatric research since the early 1960s have dramatically improved child health. The assertion that children are less healthy than they were >60 years ago is misleading and has the chilling potential to both erode public confidence in the many advances in pediatric medicine, outlined below, and devalue the need for continued investment in biomedical research that addresses the unique needs of children.

The past decades have seen seminal advances in pediatric science that have transformed child health,^9^ as demonstrated by changing patterns in the leading causes of death in childhood (Table 2). Life expectancy has risen sharply for conditions from cystic fibrosis to congenital heart disease, driven by new therapies and enhancements in surgical capacity.^10^ Research and innovation have led to improvements in childhood mortality and overall health in many ways—examples include infectious diseases prevention and treatment, prematurity care, cancer survival rates, and the dual innovations of both bone marrow and solid organ transplantation. A major transformation in child health is exemplified by enhanced neonatal intensive care for infants born prematurely, where the rate of infant deaths has decreased from 26 deaths/1000 live births in 1960, to 5.4/1000 in 2020.^11,12^ Indeed, the unfortunate death in 1963 of the newborn cousin of HHS Secretary Kennedy would be highly unusual today with advances in neonatal critical care,^13^ in particular the development of surfactant therapy that has dramatically changed the prognosis for newborn respiratory distress syndrome.^14^ Childhood leukemia was often a death sentence in 1960: today children with the diagnosis of acute lymphoblastic leukemia have a 94% five-year survival rate (https://ourworldindata.org/childhood-leukemia-treatment-history). These seminal changes in the therapy of (and prognosis for) what once were untreatable childhood diseases have changed the face of care. Compared to the 1960s, much of pediatric health care today is focused on children with medical complexity (CMC).^15^ Our successes have ensured a future for children that a generation ago would have suffered early-life mortality. Any comparison of the health of children in the US today compared to the 1960s must acknowledge this fact.

**Table 2:** Causes of pediatric mortality in the US, 1960 and 2020.

Number of deaths per 100,000 children ages 1–14, 1960^¶^	Number of deaths per 100,000 children ages 1–19, 2020^a^
1. Low birth weight/prematurity	1. Firearms (gun violence)
2. Congenital anomalies	2. Accidental deaths
3. Pneumonia and influenza	3. Drug overdose
4. Accidental deaths	4. Cancer
5. Cancer	5. Suffocation

^¶^https://www.cdc.gov/nchs/data/vsus/VSUS_1960_2B.pdf

^a^America’s Health Rankings analysis of U.S. Department of Health and Human Services, Centers for Disease Control and Prevention, National Center for Health Statistics, Multiple Cause of Death by Single Race Files via CDC WONDER Online Database, United Health Foundation, AmericasHealthRankings.org, accessed 2025).

## The changing face of child health care and emerging priorities: we must not let misinformation drive decision-making

**Fig. 1:**
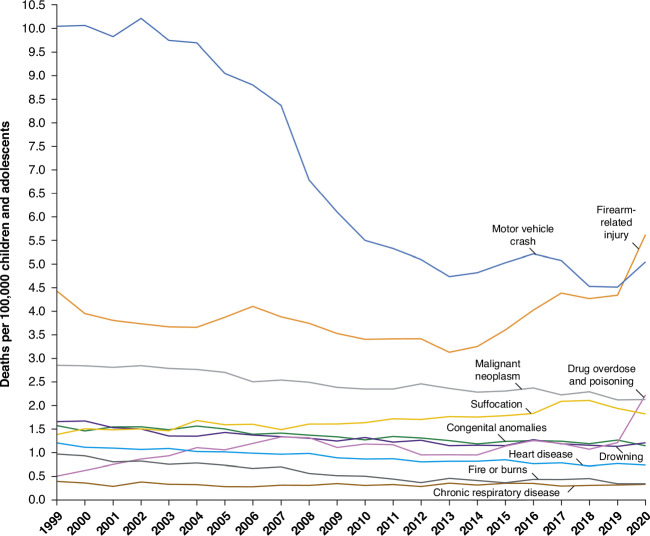
Causes of pediatric mortality in US, 2020. Reproduced from Goldstick et al, 2022, New Engl J Med^18^ with permission and approval for reproduction pending. Since 2019, firearm-related injuries have supplanted motor vehicle crashes as the leading cause of childhood death in the US.

The improvement in childhood mortality over the past decades has been offset more recently by an increase in the all-cause mortality rate for children ages 1 to 19 years between 2019 and 2022.^16,17^ Several factors are at play. The causes of childhood death have changed substantially in the past 60 years. In 1960, leading causes of childhood death included accidents/unintentional injury; complications of prematurity/low birth weight; congenital anomalies; respiratory infections; and cancer^11,12^ (Table 2). Today, the MAHA report correctly acknowledges the impact of mental health issues, sedentary lifestyles, obesity, increased use of processed foods, and increased exposures to environmental toxins and pesticides as issues of concern. However, other causes pose even more immediate and severe threats to child health. Goldstick et al. noted in 2022 the sharp increases, in children and adolescents, in firearm-related deaths and in deaths caused by drug overdose and poisonings (Fig. 1). Unintentional and intentional injuries have now emerged as leading causes of death (Table 2), driven by gun violence, motor-vehicle crashes and substance abuse.^18^ Indeed, children in the US are 15 times more likely to die by firearms than children in other high-income countries.^4^ Importantly, these aggregate data do not highlight how different demographic groups bear disproportionate risk of pediatric morbidities or the systemic factors that have contributed to child health inequities.^9,19^ Another recent analysis of overall child mortality in the US compared to high-income countries (the Organisation for Economic Co-operation and Development [OECD]), spanning 2007–2023, found that 1- to 19-year-old children and youth in the US were 80% more likely to die than their OECD counterparts: once again, the differences in mortality were driven by gun violence, motor-vehicle crashes and substance abuse.^4^ Given that these factors are the key drivers of a decline in childhood health outcomes, appropriate attention must be devoted to strategies that address these issues as any part of a comprehensive MAHA strategy.

The evolving challenges in child health in the US do not reflect a failure in past research initiatives, but they do serve notice that priorities have evolved. The emphasis in the MAHA report on the roles of chemicals and processed foods on child health is laudable, but this over-emphasis on less critical threats to child health runs a risk of “failing to see the forest for the trees”. There is unquestionably an impact of processed foods on child health,^7^ but the long-term impact of processed foods and their association with some diseases, such as type II diabetes and colorectal cancer, demonstrates, at best, weak relationships, and inconsistent input evidence.^6^ The need for further research is clear, but care must be taken not to over-state the importance of health concerns engendered by, for example, processed foods against the backdrop of several substantially greater threats to child health. Similar care must be taken with over-attribution of chemical exposures to the perceived decline in child health: for example, debates about the urgency of removing fluoride from drinking water supplies need to acknowledge the lack of evidence that the current recommended concentration of fluoride in drinking water—0.7 mg/l—poses any threat to the overall health (including the neurodevelopmental outcomes) of children.^20^ Misinformation about the risks of fluoridation must be countered by evidence, as well as the acknowledgment that the reduction in dental caries conferred by fluoridation provides a myriad of clear benefits to child health.^21^

## Misinformation and disinformation about vaccines: the most critical threat to child health

Perhaps the most concerning elements of misinformation and disinformation confronting pediatrics today are the false assertions that childhood immunizations have contributed to the perceived decline in the overall health status of children in the US. The data from decades of vaccine discovery, development and implementation speak otherwise. In children born between 1993 and 2024, the routine childhood immunization schedule in US children has prevented >500,000,000 illnesses; approximately 32,000,000 hospitalizations; and >1,000,000 deaths.^22^ Concerns about the negative impact of vaccines on child health center around a variety of factors, including the perceived risk of “chemicals” (adjuvants such as aluminum salts, thimerosal, and preservatives) and the alleged negative impact of immunization on a child’s immune system. Many of these concerns are examples that originate in disinformation, since the false statements about vaccine safety are commonly tied to an intent to deceive, driven by financial interests.^23^ There are currently two particularly disturbing elements of disinformation: 1) the concept that there is an unhealthy “immune system overload” associated with the routine childhood vaccine series; and 2) the concerns over the negative health consequences of "chemicals" in vaccines, such as the putative neurotoxicity of thimerosal, a vaccine preservative, and the disproven (but reputed) risks of the toxicities of aluminum when used as vaccine adjuvants.

The concern that the increase in the number of routinely administered childhood vaccines is linked to an increase in chronic childhood disease is contradicted by an examination of the history of the evolving vaccine schedule over the past several decades. This assertion clearly misinforms regarding scientifically proven facts about the vast diversity of immune responses that can be engendered by the human immune system.^24^ More significantly, the routine childhood immunization series in 2025 exposes the immunized child to substantially *fewer* antigens than did the standard vaccine series in 1960.^25^ There is no evidence—indeed, no biological plausibility—to the statement that the currently recommended vaccine series results in an “overload” or “weakening” of the immune system.^26^

Similar misinformation has unfortunately been promulgated regarding the putative risk of chemical components in vaccines. The use of the ethyl mercury-based preservative thimerosal was discontinued in childhood vaccines in 2001, but was until recently utilized in some vaccine preparations that were packaged in multi-dose vials. The alleged neurotoxic effects of thimerosal have been rigorously disproven in multiple studies over the past 20 years and there is no evidence linking its use to autism spectrum or neurodevelopmental disorders^27^. However, the newly reconstituted Advisory Committee on Immunization Practices recently recommended the complete elimination of the use of this agent from multi-dose vials of influenza vaccine. In the short term, this decision could potentially impact vaccine accessibility, as it may lead to exclusive use (with attendant increased costs and reduced availability) of single-dose vaccine vials. From a broader perspective, this decision may set the stage for a misinformation-driven removal of another category of “chemicals”—aluminum-based vaccine adjuvants (referred to broadly as “alum adjuvants”). Alum-based adjuvants have been widely used in vaccines for over 70 years. They are safe, well-tolerated, and effective at augmenting immune responses to vaccine antigens.^28^ A recently reported study followed more than 1.2 million children born between 1997 and 2018 for 8 years to assess the risk of developing several chronic health conditions, including asthma, allergies, neurodevelopment disorders, and autoimmune disorders. No association with alum-based adjuvants used in pediatric vaccines was found for any of these health conditions.^29^ However, misinformation and disinformation about the safety of alum continue to be expressed, leading to the unusual request by the US HHS Secretary (refused by the journal's editor) that the study be retracted (https://www.nature.com/articles/d41586-025-02682-9).

## Rejecting the allure of misinformation and disinformation should not be a partisan choice

Misinformation and disinformation lead to misperceptions about the current challenges to child health in the US. The causes of the embracement of misinformation and disinformation are diverse and incompletely understood. Social media clearly plays a role,^30,31^ as do political affiliations,^32^ distrust of authority figures,^33^ and a lack of basic medical knowledge.^34^ It must also be acknowledged that there is a need to improve our current health system, which leaves many families unable to access care or establish a trusted medical home.^35^ Irrespective of the causes, the consequences can be devastating. Vaccination rates in children are in a state of steady decline in the US,^36,37^ and falling rates of coverage have contributed to two pertussis deaths in unimmunized infants in Kentucky (https://publications.aap.org/aapnews/news/32374/2-Kentucky-infants-die-of-pertussis-as-cases-rise?autologincheck=redirected). Two infant deaths from pertussis (with undisclosed vaccination status), as well as a surge in pertussis-related hospitalizations, have recented been reported in Louisiana (https://publications.aap.org/aapnews/news/31750/2-Louisiana-infants-die-of-pertussis-as-infections). Re-emergence of other vaccine-preventable diseases have been reported, such as polio^38^, and a major outbreak of measles^39^ has similarly been observed. The current measles epidemic by mid-2025 had already exceeded the previous record for the highest total number of cases since the disease was (temporarily) eliminated from circulation in the US in 2000.^40^

Medical misinformation and disinformation impact child health unevenly and inequitably. An example is the 2017 outbreak of measles in a Somali community in Minnesota.^41,42^ A study from the Health Information National Trends Survey (HINTS) examined the impact of health-related misinformation accessible on social media, exploring the way in which racial, ethnic, and sociodemographic factors impacted susceptibility to misinformation.^43^ Disparities across racial, age, and income groups were noted, underscoring a need to tailor interventions for the specific patient populations being evaluated.

Recognition that confronting misinformation and disinformation is a key priority for child health should not be a partisan issue. Although we recognize the impact of current political and social realities in the US, we emphasize that all examples of misinformation and disinformation are problematic for child health, regardless of their origin. Misinformation and disinformation harm children; endanger patient-physician relationships that are critical to optimizing the care of children; and arise from all sides of the political spectrum. Examples across the political and ideological spectrum include misinformation and disinformation downplaying the major health risks and disparaging medical management of obesity in children (what has been referred to as “obesity politics”)^44^, and in the endorsement of unproven therapies in academic settings for treatment of autism spectrum disorders.^45^ Misinformation and disinformation must be recognized, acknowledged and corrected regardless of the ideologic origin of that information.

## Strategies to confront misinformation and disinformation

As pediatricians, whether in clinical practice, administration, or research, it is a time for introspection. How do we begin to confront the misinformation and disinformation that threatens the health of American children? Here are some suggestions:

1. Continued advocacy for investment in basic, clinical and translational science research in pediatrics. It is vital that we build on past successes and be intentional and proactive about communicating and celebrating these advances.
2. Emphasis on the pivotal role of the primary care provider. Pediatricians and other primary care physicians are historically highly trusted individuals,^46^ although there is evidence that the level of trust declined during the COVID-19 pandemic.^47^ Academic societies and institutions should continue their work on developing educational and training modules that empower primary care physicians to address misinformation and disinformation.
3. Coordinated advocacy by medical societies. The National Academy of Sciences, Engineering and Medicine released a statement earlier this year (https://www.nationalacademies.org/news/2025/03/america-cant-be-great-without-great-science-that-is-where-the-academies-can-help) warning that the risk of dismantling the American research infrastructure built over many decades imperils the lives of both children and adults. Leaders of pediatric and adult medical organizations must continue to present a unified and strong opposition against indiscriminate and draconian cuts to medical research and to studies that support evidence-based medicine.
4. Improved science education. Individuals who gain knowledge about a health topic are less likely to endorse or believe misinformation on that subject.^34^ We recommend expansion, development and implementation of educational programs that target middle school students, high school students, and undergraduates, with a goal of engaging students in scientific discovery early in their educational pathways. One successful model of this approach was the APS-SPR Medical Student Research Program.^48^ Acquisition of a working knowledge of scientific principles early in the educational process will enhance the ability of an individual to discern the validity of information throughout their lifetime. For health professionals, medical schools should incorporate modules on dealing with medical misinformation and disinformation into the core curriculum.^47^
5. Increased emphasis on a family’s lived experiences, particularly as related to vaccine-preventable diseases and their experiences that have benefitted the care of their children for diseases that used to cause early-life mortality. Families sharing their personal experiences can help emphasize the positive impact of child health research on “real-world” outcomes and fight the spread of misinformation (https://apnews.com/article/vaccines-measles-polio-whooping-cough-rubella-af4cd1aef8f408a960601df6372f9c32). Family engagement and leadership can also help to identify and implement patient and family centered improvements in our nation’s health care system. Interdisciplinary and cross-sector collaborations (with schools, public health agencies, community organizations) are a vital area for emphasis. Such approaches must also connect with diverse communities and cultures, given the disproportionate impact that misinformation and disinformation have on these families.
6. Even in a climate of extreme partisanship, “across-the-aisle” consensus agreements are possible. An example was a legislative initiative to commence newborn screening for congenital cytomegalovirus infection, co-sponsored by both major political parties and overwhelmingly adopted by a state legislature in 2021.^49^ Legislators and public health policy-makers should endeavor to embrace consensus in addressing the key priorities of child health, including the shared goal of minimizing the impact of misinformation and disinformation. Innovative, non-partisan and non-confrontive strategies are needed to address this problem, and to fully acknowledge the root causes and the systemic factors that have contributed to the decline in child health and to the increase in chronic child health conditions, as described by the National Academies of Sciences, Engineering, and Medicine and the National Research Council.^16,50^

In summary, we have realized enormous progress through basic, clinical and translational research that has dramatically transformed pediatric care over the past several decades. Misinformation and disinformation threaten this progress and strategies to address this issue are needed. Pediatricians should pursue strength-based approaches that call on society to work together, build on what we know, and continue new discovery to improve child health. In our view, some of the best approaches to confronting misinformation and disinformation in medicine include promoting objective, data-driven examples of the spectacular successes in pediatric discovery over the past half-century, acknowledging the key and emerging threats to child health we confront today, and empowering new research that addresses these evolving priorities.
